# Vascular endothelial growth factor (VEGF) in leptomeningeal metastasis: diagnostic and prognostic value

**DOI:** 10.1038/sj.bjc.6601953

**Published:** 2004-06-22

**Authors:** U Herrlinger, H Wiendl, M Renninger, H Förschler, J Dichgans, M Weller

**Affiliations:** 1 Department of General Neurology, Hertie Institute for Clinical Brain Research, University of Tübingen, Hoppe-Seyler-Str. 3, D-72076 Tübingen, Germany

**Keywords:** VEGF, leptomeningeal metastasis, prognostic factor, CSF

## Abstract

This study examined the diagnostic and prognostic value of vascular endothelial growth factor (VEGF) levels in the cerebrospinal fluid (CSF) of 39 patients with leptomeningeal metastasis (LM). Vascular endothelial growth factor levels at diagnosis were significantly higher in patients with LM (median 359 pg ml^−1^) than in patients with other neurological diseases (median <25 pg ml^−1^). The specificity of VEGF levels above 250 pg ml^−1^ for LM was high (98.3%), while the sensitivity was low (51.4%; 73% for VEGF values above 100 pg ml^−1^). In 49% of the LM patients, particularly with lymphoma or medulloblastoma, VEGF levels were below 250 pg ml^−1^ and thus in the range of VEGF levels in other neurological diseases. Vascular endothelial growth factor levels correlated significantly with CSF lactate and albumin. Vascular endothelial growth factor levels mirrored the clinical course with a marked reduction in response to therapy and an increase at relapse in some patients who had serial CSF samples available. Multivariate Cox regression analysis showed VEGF below 100 pg ml^−1^ (relative risk (RR)=4.24, *P*=0.0002) and age below 60 years (RR=2.5, *P*=0.004) to be associated with longer survival in LM. In conclusion, CSF VEGF levels in LM vary considerably. High VEGF levels have a very high specifity for LM and may help to establish the diagnosis. The role of VEGF as a predictor of outcome should be substantiated in prospective studies.

The aggressive treatment of leptomeningeal metastasis (LM) with intrathecal or systemic chemotherapy or radiotherapy should require the unequivocal demonstration of tumour cell dissemination within the subarachnoid space. Malignant cells in cerebrospinal fluid (CSF) and contrast-enhancing leptomeningeal lesions on neuroimaging, suggestive of tumour cell deposits, are regarded diagnostic of LM. In any other situation suggestive, but not diagnostic, of LM, additional CSF parameters, would be helpful for establishing the diagnosis. Ideally, such parameters would not only be of diagnostic, but also of prognostic value, thus helping to choose the adequate therapy. Apart from the fact that elevated CSF protein was almost uniformly found as a negative prognostic factor, the literature remains controversial on the prognostic value of low CSF glucose or high CSF lactate levels or high age (Boogerd *et al*, 1991; Balm and Hammack, 1996; Fizazi *et al*, 1996). The many attempts at defining additional diagnostic or prognostic CSF parameters for LM have met with little success, mostly because of low specificity. Vascular endothelial growth factor (VEGF), a strong inducer of angiogenesis and possibly a cellular survival factor in many types of cancer, has been evaluated in one preliminary study: VEGF levels were uniquely high in 11 patients with LM, while VEGF remained low in patients with non-neoplastic neurological diseases (Stockhammer *et al*, 2000). The present study assesses the differential diagnostic value of CSF VEGF levels in a substantially larger population with a broader spectrum of primary tumours. Furthermore, VEGF was investigated as a prognostic parameter in LM, thus extending the scope of application for the measurement of VEGF in LM.

## PATIENTS AND METHODS

A total of 39 patients diagnosed and treated for LM at the University of Tuebingen Medical Center between 1995 and 2002 were included in this retrospective study. Cerebrospinal fluid samples from the time of diagnosis of LM, before therapy was initiated, were available in 37 of these patients. Two additional patients had multiple samples withdrawn during therapy but no initial diagnostic sample. Cerebrospinal fluid was routinely drawn by the lumbar route at diagnosis, whereas follow-up samples, available in 10 patients, were usually collected from ventricular reservoirs. In total, 50 patients with non-neoplastic neurological diseases and normal values for CSF cell count, albumin and lactate, 28 patients with multiple sclerosis, and 37 patients with presumed infectious CNS disease were included for comparison. The patients in the latter group were randomly picked from our 2002 and 2003 database if infectious CNS disease was suspected clinically and CSF cell counts or albumin were abnormal. They presented with clinical symptoms and signs such as seizures, stiff neck, focal neurological symptoms including hemiparesis, ataxia, cranial nerve palsies or (poly)radicular symptoms, and altered mental status. Only one of these patients had a history of systemic tumour (colon carcinoma). The 37 patients with suspected infectious disease finally received the following diagnoses: 11 acute or subacute bacterial meningitis or ventriculitis; six acute (poly)radiculitis, due to borreliosis (*n*=4) or a cause not further specified (*n*=2); 12 acute lymphocytic meningitis or meningoencephalitis, due to varicella zoster infection (*n*=5), tick-borne encephalitis virus (*n*=3) or a cause not further specified (*n*=4); three chronic meningitis or meningoencephalitis, due to presumed tuberculosis (*n*=2) or syphilis (*n*=1); five patients were eventually not confirmed to have infectious CNS disease. In the patients with LM, the diagnosis was proven by the demonstration of neoplastic cells in the CSF (49%) or contrast-enhancing subarachnoid tumour cell deposits on MRI (10%) or both (41%). Survival was recorded in all patients. Data on systemic tumour control at the time of diagnosis of LM and the prevalence of subarachnoid contrast-enhancing lesions were available in 37 out of 39 patients. Information on clinical response to therapy (improvement, no change, or worsening of neurological status) as graded by the treating physician was available for 23 out of 39 patients.

Standard CSF parameters such as cell counts and levels of albumin, IgG, and lactate were determined at the time of diagnosis of LM. The presence of malignant cells in CSF was analysed on cytospin preparations. Cerebrospinal fluid (500 *μ*l) was centrifuged for 4 min at 1100 r.p.m., and the slides were air-dried, fixed with acetone and stored until staining at −20°C. After Pappenheim staining, CSF cells were evaluated by cytological criteria. The origin of malignant cells from particular primary tumours was further analysed using immunocytochemistry on additional cytospin preparations. Tumour cells were not quantified. For VEGF analysis, aliquots of CSF samples were centrifuged for 10 min at 4°C and the supernatant was stored at −80°C. Vascular endothelial growth factor levels were determined by ELISA (R&D Systems, Minneapolis, MN, USA) in duplicate with 50 *μ*l CSF per well and VEGF standard dilutions (15.6–2000 pg ml^−1^) as provided by the manufacturer. Antibody binding was visualised by tetramethylbenzidine and hydrogen peroxide and the colour reaction was measured at 450 nm. To correct for imperfections of the plate, another measurement was carried out at 540 nm. The intraassay and interassay variation of this assay was below 10%. The detection limit of the assay was 25 pg ml^−1^. The assay recognises both the 121 and 165 kDa VEGF isoforms. According to the manufacturer's brochure, there are no known significant interactions of related proteins with the VEGF measurement. At high concentrations, recombinant human VEGF receptor 1 (>1250 pg ml^−1^) and 2 (>10 000 pg ml^−1^) and mouse VEGF receptor 2 (>10 000 pg ml^−1^) may interfere with the VEGF assay. The sensitivity, specificity, positive and negative predictive value and accuracy of VEGF as a marker for LM was estimated using different cutoff levels. Cerebrospinal fluid VEGF values of LM patients at diagnosis were correlated with standard CSF parameters using Pearson product moment correlation. Differences in CSF parameters between subgroups of patients were analysed for significance using the Mann–Whitney *U*-test. Survival was determined according to Kaplan and Meier (1958). The association of dichotomised CSF (standard and VEGF) and clinical parameters (age, gender, presence of subarachnoidal contrast-enhancing lesions on MRI) with survival was analysed in univariate and multivariate Cox regression analysis. The course of VEGF levels in response to therapy was followed in relation to the clinical course in 10 patients with multiple follow-up CSF samples.

## RESULTS

### Increased CSF VEGF levels in LM

Initial VEGF values in 37 patients with LM are summarised in Table 1

**Table 1:** CSF VEGF levels in 37 patients at the time of diagnosis of LM and in 115 patients with other neurological diseases

	***N***	**Median VEGF value (pg ml^−1^)**	**Minimal VEGF value (pg ml^−1^)**	**Maximal VEGF value (pg ml^−1^)**	**Pat. <100 pg ml^−1^**	**Pat. >250 pg ml^−1^**
LM (all primary tumours)	37	359	<25	>3000	10/37 (27%)	19/37 (51%)
						
Brain tumours	13	123	<25	>3000	4/13 (31%)	5/13 (38%)
	8 glioblastoma					
	2 anaplastic astrocytoma					
	1 anaplastic oligodendroglioma					
	2 medulloblastoma					
Lympho-reticular tumours	3	<25	<25	<25	3/3 (100%)	0/3 (0%)
	2 CLL					
	1 Langerhans' histiocytosis					
Melanoma	7	377	<25	>3000	2/7 (28%)	5/7 (72%)
Breast cancer	5	1721	<25	>3000	1/5 (20%)	4/5 (80%)
Lung cancer	4	497	92	>3000	1/4 (25%)	2/4 (50%)
Other solid primary tumours	5	776	130	>3000	0	3/5 (60%)
	4 CUP					
	1 ovarian cancer					
						
Other neurological diseases, CSF normal	50	<25	<25	180	48 (96%)	0 (0%)
Multiple sclerosis	28	<25	<25	<25	28 (100%)	0 (0%)
(Suspected) infectious diseases, pathological CSF	37	<25	<25	2116	31 (84%)	2 (5%)

CLL=chronic lymphocytic leucaemia; CUP=cancer of unknown primary; CSF=cerebrospinal fluid; LM=leptomeningeal metastasis; VEGF=vascular endothelial growth factor.

. The median CSF VEGF concentration was 357 pg ml^−1^ in the entire group and 569 pg ml^−1^ (range <25 to >3000 pg ml^−1^) in patients with systemic primary tumours excluding primary brain tumours. Vascular endothelial growth factor levels above 250 pg ml^−1^ were frequently found in LM from breast cancer and melanoma (Table 1). No VEGF was detected in the CSF of three patients with LM from lymphoreticular tumours. Vascular endothelial growth factor levels in patients with LM from primary brain tumours varied. Vascular endothelial growth factor levels were around 100 pg ml^−1^ in medulloblastoma patients, but were distributed over the whole range of measurements in glioma patients.

Vascular endothelial growth factor levels did not differ between patients over 60 years (*n*=12; median 726 pg ml^−1^) and patients up to 60 years of age (*n*=25; median 146 pg ml^−1^; *P*=0.48), patients with systemic tumour cell deposits at the time of diagnosis of LM (*n*=25; median 377 pg ml^−1^) and patients without (*n*=10; median 174 pg ml^−1^; *P*=0.21), or patients with tumour manifestations in the brain or dura (*n*=25; median 377 pg ml^−1^) and patients without (*n*=11; median 241 pg ml^−1^; *P*=0.35). Also, there was no difference in CSF VEGF values between patients with subarachnoid contrast-enhancing lesions detected by MRI (*n*=18; median 556 pg ml^−1^) and patients without such lesions (*n*=18, median 125 pg ml^−1^, *P*=0.32). Further, the VEGF levels of patients diagnosed by MRI only but not by detection of tumour cells in CSF (*n*=4; median 195 pg ml^−1^) did not significantly differ from the patients with tumour cells detectable in CSF (*n*=33; median 359 pg ml^−1^; *P*=0.22). Cerebrospinal fluid VEGF levels were highly correlated with lactate levels (*n*=34; *r*=0.79; *P*<0.00001) and less so with CSF albumin as a marker for blood–brain barrier disruption (*n*=37; *r*=0.54; *P*=0.0005), but not with CSF cell counts (*n*=36; *r*=0.27; *P*=0.16).

### Diagnostic value of VEGF in LM

Vascular endothelial growth factor was measured in the CSF of 115 patients with presumed non-neoplastic neurological disease. One group of 50 patients included patients with normal CSF cell counts, albumin, IgG, and lactate who had lumbar puncture during myelography or during diagnostic work-up to exclude inflammatory CNS diseases. Only two of these 50 patients had values slightly above 100 pg ml^−1^: one patient with symptomatic complex-partial seizures from a previous stroke and one patient with cerebellar ataxia of unknown cause. All of 28 MS patients had VEGF levels below detection limit (25 pg ml^−1^). A third group included 37 patients with pathological CSF due to (suspected) infectious disease. Six of these patients had VEGF levels above 100 pg ml^−1^: three patients with VEGF values between 111 and 229 pg ml^−1^ had ventriculitis, one patient (198 pg ml^−1^) had zoster meningitis, one patient (441 pg ml^−1^) had a diffuse, nonenhancing swelling of a cerebellar hemisphere of unknown origin. Infectious causes were discussed but with a history of active colon cancer a neoplastic cause could not be ruled out. Another patient had exorbitantly high levels of VEGF in the CSF (2116 pg ml^−1^). This patient had axonal polyneuropathy and a suprasellar lesion of unknown aetiology, but no evidence of LM by MRI or CSF cytology. Sensitivity, specificity, positive and negative predictive value, and accuracy for CSF VEGF as a diagnostic marker in LM are summarised in Table 2

**Table 2:** Diagnostic value of CSF VEGF levels in patients with LM

**Cutoff value**	**Sensitivity (%)**	**Specificity (%)**	**Positive predictive value (%)**	**Negative predictive value (%)**	**Accuracy (%)**
100 pg ml^−1^	73	93	77.1	91.5	88.2
250 pg ml^−1^	51.4	98.3	90.5	86.3	86.8

CSF=cerebrospinal fluid; LM=leptomeningeal metastasis; VEGF=vascular endothelial growth factor.

. With a cutoff level of 250 pg ml^−1^, specificity and positive prognostic value were excellent, whereas sensitivity was low. With a less rigorous cutoff level of 100 pg ml^−1^, the sensitivity increased (73.7%) while the positive prognostic value was relatively low (77.8%).

### Prognostic value of VEGF in LM

Vascular endothelial growth factor levels at the time of diagnosis of LM were also evaluated for their prognostic value for a clinical response to therapy (radiotherapy, chemotherapy, or combined radiochemotherapy) and overall survival. Pretherapy VEGF values in eight patients with improvement of neurological status upon therapy (median 768 pg ml^−1^) were not significantly different from VEGF levels of 15 patients who did not improve (median 241 pg ml^−1^; *P*=0.48). Cerebrospinal fluid cell counts (*P*=0.73), albumin (*P*=0.65), IgG (*P*=0.94), or lactate (*P*=0.31) did not show significant differences between patients with and without clinical responses either.

According to a univariate Cox regression analysis (Table 3

**Table 3:** Prognostic factors in patients with LM

	**Median survival in months (number of patients)**		**Risk ratio (lower–upper 95% CI)**	
	**Condition 1**	**Condition 2**	**Condition 2 *vs* condition 1**	***P***
*Univariate analysis*				
Age	⩽60 years	>60 years		
	5.8 (26)	1.7 (11)	**1.89 (1.13–3.21)**	**0.02**
				
Gender	Female	Male		
	6.8 (19)	2.4 (18)	1.14 (0.74–1.72)	0.58
				
CSF cell count	Normal	Elevated		
	15.9 (12)	4.4 (24)	1.28 (0.80–2.17)	0.31
				
CSF albumin	Normal	elevated		
	NA (7)	3.6 (30)	1.74 (0.93–4.36)	0.08
				
Subarachnoid contrast-enhancing lesions	No	Yes		
	5.6 (19)	4.4 (18)	0.83 (0.52–1.30)	0.42
				
CSF VEGF	⩽100 pg ml^−1^	>100 pg ml^−1^		
	NA (10)	2.4 (27)	**3.54 (1.6–15.0)**	**0.0003**
				
*Multivariate analysis (n*=*36)*				
Age	⩽60 years	>60 years	**2.50 (1.34–4.87)**	**0.004**
				
Gender	Female	Male	1.61 (0.9–3.1)	0.11
				
CSF cell count	Normal	Elevated	0.63 (0.32–1.24)	0.18
				
CSF albumin	Normal	Elevated	1.22 (0.56–3.31)	0.63
				
Subarachnoid contrast-enhancing lesions	No	Yes	0.85 (0.52–1.41)	0.53
				
VEGF in CSF	⩽100 pg ml^−1^	>100 pg ml^−1^	**4.24 (1.77–18.7)**	**0.0002**

NA=not assessable; LM=leptomeningeal metastasis; CSF=cerebrospinal fluid; VEGF=vascular endothelial growth factor. Bold values highlight the prognostic factors that showed significant effects on media survival.

), a CSF VEGF level above 100 pg ml^−1^ at diagnosis was a strong predictor of poor survival. Age over 60 years at diagnosis was a less potent, but still significant negative predictor of survival. Other CSF parameters had no prognostic value but due to the small sample size less potent prognostic factors may have remained below detection level. Both prognostic factors found in the univariate analysis were confirmed in the multivariate analysis (Table 3). The CSF VEGF level was also found as a prognostic factor (relative risk (RR) 2.51; 95% confidence interval 1.12–10.8, *P*=0.021) when the univariate analysis was restricted to the 24 patients with systemic primary tumours.

### Sequential VEGF determinations in the follow-up of LM patients

A total of 10 patients had sequential CSF samples available for follow-up VEGF determinations. Eight of these patients had already VEGF measurement at diagnosis. Two more patients without initial CSF samples but multiple samples during therapy were included, one male with adenocarcinoma of the lung and one female with breast cancer.

**Figure 1:**
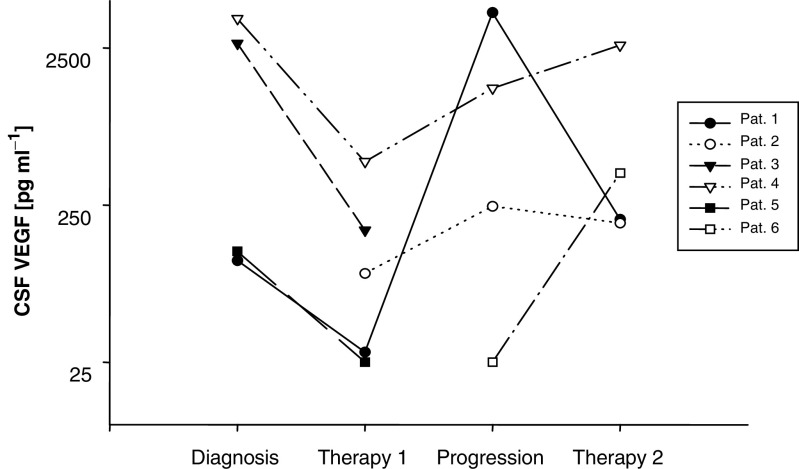
Time course of CSF VEGF levels during the course of disease in six patients with multiple samples. Patient 1 had oligodendroglioma and received PCV chemotherapy as first-line therapy and craniospinal radiotherapy as second-line therapy; patient 2 had adenocarcinoma of the lung, received intrathecal MTX as first-line and intrathecal thiotepa as second-line therapy; patient 3 had breast cancer and received intrathecal MTX and craniospinal radiotherapy; patient 4 had melanoma and received nitrosourea-based systemic chemotherapy and intrathecal MTX as first-line and intrathecal AraC as second-line therapy; patient 5 had squamous carcinoma of the lung and received systemic chemotherapy with etoposide and carboplatin plus intrathecal MTX therapy; patient 6 had breast cancer and received intrathecal MTX and whole brain radiotherapy as first-line therapy and intrathecal thiotepa as second-line therapy.

The course of six patients with CSF VEGF values changing upon therapy and relapse is shown in Figure 1

. Four additional patients did not show any change of VEGF levels in multiple CSF samples and were not included in Figure 1. Two patients (VEGF <100 pg ml^−1^) received systemic nitrosourea therapy (one melanoma, one glioblastoma) and remained neurologically stable; one patient (VEGF <100 pg ml^−1^) received intrathecal MTX (one chronic lymphocytic leukaemia). Her neurological status worsened upon therapy. Vascular endothelial growth factor levels of about 400 pg ml^−1^ remained unchanged during intrathecal MTX therapy in one patient with melanoma although his neurological status worsened.

Four of six patients plotted in Figure 1 (one oligodendroglioma, patient (P) 1; one breast cancer, P3; one melanoma, P4; one lung cancer P5) were evaluable for the effects of primary intrathecal or systemic chemotherapy with or without radiotherapy on CSF VEGF. In all four patients, CSF VEGF levels were substantially reduced upon therapy and these patients experienced a partial (*n*=2) or complete (*n*=2) resolution of clinical symptoms; three patients were also evaluable for a cytological response: one patient with oligodendroglioma showed CSF clearance of malignant cells, in two patients CSF was not cleared. Three of six patients (P1, P2, P4) were evaluable for CSF VEGF levels upon relapse. In all three patients, VEGF levels increased substantially. Four of six patients (P1, P2, P4 P6) were evaluable for response of VEGF levels upon second-line therapy. Vascular endothelial growth factor levels fell again in response to (secondary) craniospinal radiotherapy in a patient with oligodendroglioma. This patient also showed improvement of neurological status. In a patient with lung cancer, second-line intrathecal thiotepa therapy led to a stabilisation of VEGF values and improvement of neurological status. In two other patients (one melanoma, one breast cancer), VEGF levels increased and neurological status declined.

## DISCUSSION

The present study demonstrates the diagnostic value of CSF VEGF determinations in patients with LM. We illustrate the high specificity of elevated VEGF levels for LM (Table 2), the prognostic power of initial VEGF values for survival (Table 3) and the possible use of CSF VEGF as a parameter to monitor response to therapy (Figure 1). Nevertheless, this study also highlights that neoplastic and non-neoplastic leptomeningeal disease cannot be safely distinguished by CSF VEGF and that normal VEGF levels do not rule out LM.

A preliminary study with 11 LM patients (Stockhammer *et al*, 2000) had reported that CSF VEGF levels distinguish between neoplastic and non-neoplastic disease since the ranges of value obtained in these two groups did not overlap. In the present study with a substantially larger patient population (*n*=37), the differences in VEGF values between the two groups of patients were less clearcut. The VEGF values were still highly significantly different in the two groups, but the ranges overlapped (Table 1). Some LM patients (20%) had VEGF levels below or close to the detection limit. The present study included a much broader spectrum of primary tumours while the previous study comprised patients with breast, lung, and ovarian cancer only (Stockhammer *et al*, 2000). Here also patients with primary brain tumours (e.g. medulloblastoma), melanoma, lymphoma, and cancer of unknown primary (CUP) were included. Patients with lymphomas, particularly CLL, or medulloblastomas had low VEGF levels. Even in some patients with glioblastoma, VEGF levels remained low. There may possibly be some differences in VEGF levels between LM from different primaries. However, besides the low VEGF levels in patients with lymphoreticular tumours, differences in VEGF levels between different tumour entities were not sufficiently prominent to be of differential diagnostic relevance. The finding of low VEGF levels in a proportion of patients with LM greatly reduces the sensitivity of the assay to detect LM. However, since most cases of LM can be diagnosed by CSF cytology or MRI, sensitivity is not the most important feature of a VEGF assay for LM. Specificity is more important since such an assay may allow to detect high levels of VEGF in the CSF of patients without diagnostic CSF or MRI findings. Only two of 115 control cases had VEGF levels above a cut–off level of 250 pg ml^−1^ (Table 1). Of note, the composition of the control group influences the figures for specificity and sensitivity of VEGF in the diagnosis of LM: if the control group was restricted to patients with presumed infectious disease as the most appropriate controls, specificity at a cutoff level of 250 pg ml^−1^ would be reduced from 98.3 to 94.5%. Interestingly, parenchymal CNS metastases are not associated with increased CSF VEGF levels (Stockhammer *et al*, 2000) and thus do not impair the specificity of elevated VEGF levels for LM. Furthermore, the present study confirms that CSF VEGF levels are low in MS (Watanabe *et al*, 1998) and that slightly increased VEGF levels are found in bacterial meningitis (Stockhammer *et al*, 2000). Interestingly, one patient with zoster meningitis had slightly increased VEGF levels.

In contrast to the previous smaller series (Stockhammer *et al*, 2000), we found a strong correlation of VEGF with CSF lactate and a less stronger correlation with CSF albumin. The correlation with CSF lactate makes sense in that lactate may reflect the metabolism of tumour cells in a low-oxygen environment which in turn induces VEGF expression in tumour cells (Shweiki *et al*, 1995; Fukumura *et al*, 2001; Ziemer *et al*, 2001). In fact, acidic pH itself induces VEGF expression (Fukumura *et al*, 2001). The possible value of VEGF as a CSF marker for monitoring LM is reflected by preliminary data obtained in a small series of patients (Figure 1). Marked reductions in VEGF levels were accompanied by clinical improvement in all evaluable patients, whereas increases in VEGF levels were associated with relapse. Prospective studies will have to explore in more depth the possibility of monitoring LM by serial measurements of VEGF. Importantly, VEGF is not only a diagnostic tool, but may also have prognostic value. Here, VEGF was a much more potent prognostic factor than CSF albumin. Further prospective studies have to clarify whether CSF VEGF levels may serve for risk stratification of patients in studies with LM. The value of VEGF as a prognostic factor also corresponds to the fact that increased VEGF is also an independent negative prognostic factor in systemic tumours and solid brain tumours (Berkman *et al*, 1993; Ohta *et al*, 1996).

Many proteins have been investigated as diagnostic or prognostic CSF parameters in LM patients during the last 30 years, including carcinoembryonic antigen (CEA; Klee *et al*, 1986), lactate dehydrogenase (LDH; Seidenfeld and Marton 1979; Van Zanten *et al*, 1986), beta-glucuronidase and beta-microglobulin (Ongerboer de Visser *et al*, 1985), a combination of tissue polypeptide antigen and CK-BB (Bach *et al*, 1993), fibronectin (Weller *et al*, 1990), and the epithelial HMFG-1 antigen (Moseley *et al*, 1989). Although most of these parameters are highly sensitive for LM, their specificity is low because infectious CNS diseases also showed abnormal CSF findings or this group of patients was not studied for comparison. In contrast to VEGF (Stockhammer *et al*, 2000), the CSF levels of some biomarkers are highly influenced by their serum concentrations or may not allow to differentiate between leptomeningeal and epidural growth (Klee *et al*, 1986). Compared with these previously markers, VEGF appears to offer a favourable profile of sensitivity and specificity.

In conclusion, this report provides further evidence for the high specificity of markedly increased levels of CSF VEGF in LM. However, the relationship between malignant cells in the CSF and VEGF may be more complex than previously reported (Stockhammer *et al*, 2000) in that the absence of increased CSF VEGF does not exclude LM. With the strong evidence for VEGF as a prognostic parameter (Table 3) and the potential ability to follow LM patients with serial VEGF determinations (Figure 1), VEGF appears to be an interesting candidate for prospective evaluation in further clinical studies of LM.
